# Disease association mapping in *Drosophila* can be replicated in the wild

**DOI:** 10.1098/rsbl.2010.0329

**Published:** 2010-05-05

**Authors:** Lena Wilfert, Francis M. Jiggins

**Affiliations:** Department of Genetics, University of Cambridge, Cambridge CB2 3EH, UK

**Keywords:** association mapping, linkage mapping, replication, field, natural population

## Abstract

Association and linkage mapping have become important tools in understanding the genetics of complex traits, including diseases in humans. As the success of association mapping is reduced by small effect sizes and limited power, linkage studies in laboratory-based model systems are still heavily used. But whether the results of these studies can be replicated in natural populations has been questioned. Here, we show that a polymorphism in the gene *ref*(*2*)*P*, which had previously been linked to sigma virus resistance in *Drosophila melanogaster* under laboratory conditions, also provides resistance against the virus in female flies in a wild population in the field. This genetic association is thus upheld in spite of a known genotype-by-genotype interaction and environmental variation.

## 1. Introduction

Our understanding of the genetic basis of disease in humans has been greatly advanced in recent years by genome-wide association mapping. However, in these studies both the power to detect association and the effect size of particular polymorphisms on disease phenotypes are typically low (Altshuler *et al*. 2008). Thus, far from making them obsolete, the advances in human association mapping have exemplified the need to study the genetic basis of complex traits such as disease in tractable model systems.

By relating phenotypic and genetic variation, linkage and association mapping allow us to identify the genetic basis of complex traits (Mackay *et al*. 2009). These approaches are most powerful in model organisms, where it is possible to control the environment and genetic background, and to measure traits across many individuals with identical genotypes (Mackay *et al*. 2009). These methods have proven highly successful. Both human disease association mapping studies (i.e. Burton *et al*. 2007; Altshuler *et al*. 2008) and quantitative trait loci (QTL) studies on agricultural systems (reviewed in Wilfert & Schmid-Hempel 2008) have shown that results are, at least to some extent, repeatable across genetic backgrounds and different environmental conditions. Nevertheless, whether results from laboratory studies on model organisms are relevant in natural populations has been called into doubt (Macdonald & Long 2004; Gruber *et al*. 2007). For example, Macdonald & Long (2004) showed that a regulatory polymorphism for *hairy* that had been shown to influence bristle number in laboratory studies was not associated with this phenotype in two wild populations of *Drosophila melanogaster*. Similarly, QTLs for *Plasmodium*-refractoriness in *Anopheles gambiae* (Zheng *et al*. 1997) appear to be of little importance in the field, as the particular trait studied in the laboratory is virtually absent from wild populations (Schwartz & Koella 2001, 2002). These examples highlight the need for studies demonstrating whether laboratory-based association and linkage mapping in model systems are identifying genes that are relevant in natural populations.

The effect of a particular polymorphism is often context-dependent: first, the effect may depend on the genetic background in which it is assayed, as it may be affected by dominance or epistatic interactions with other loci; second, genotype-by-environment interactions often play an important role; and even the sex of the experimental population matters, as the magnitude and direction of effects can depend on whether a trait is assayed in males or females (Mackay *et al*. 2009). Where a trait involves biotic interactions, this uncertainty is going to be still more pronounced. A case in point is host–parasite interactions, where the effect of a polymorphism in one partner may depend on the other partner's genotype. For such interactions, it has been shown that the identity of QTLs for resistance in the host is highly variable if experiments are repeated using different isolates of a particular parasite, and this is a greater source of variation than genotype-by-environment interactions (Wilfert & Schmid-Hempel 2008).

We have studied the effect of a host resistance gene in *D. melanogaster* on transmission rates of the sigma virus in the field. The sigma virus is a natural parasite that infects up to 20 per cent of *D. melanogaster* and is transmitted vertically from parent to offspring by both the egg and sperm (Fleuriet 1988; Carpenter *et al*. 2007). Laboratory studies have found that a polymorphism in the host gene *ref*(*2*)*P* greatly reduces transmission of the virus (Bangham *et al*. 2008*a*). This gene was originally identified by linkage mapping (Guillemain 1953), and subsequently cloned and extensively studied for polymorphisms (Contamine *et al*. 1989; Dru *et al*. 1993; Wayne *et al*. 1996). An association mapping study using a panel of 84 chromosome substitution lines isolated from a natural population has confirmed that the causal polymorphism was a complex mutation, replacing the amino acids Gln and Asn with a single Gly (Bangham *et al*. 2007). Here, we ask whether this polymorphism is associated with resistance in a wild population of *D. melanogaster*.

## 2. Material and methods

We measured the rate at which males and females transmit the sigma virus to their offspring in a field population of *D. melanogaster* in Athens, Greece. Flies were collected over 5 days in October 2007 from fruit baits placed in an urban area approximately 300 m in diameter and were then placed individually in vials with maize fly medium (see the electronic supplementary material). To measure the male transmission rate, we added two uninfected virgin females to every male. These virgins were collected from an outbred laboratory population of *D. melanogaster* that we produced by mixing isofemale lines from the same population. The flies were sheltered from rain and direct sunlight but were otherwise kept under field conditions. The flies were allowed to lay eggs and were removed from the vial after 2–5 days to limit larval competition. The parental flies were then maintained on agar (2% w/v agar, 8.4% w/v sugar).

The wild flies and their offspring vials were transferred to the laboratory and tested for sigma virus infection by exposing them to pure CO_2_ at 12°C for 15 min and recording their status 2 h later. While uninfected flies quickly recover from the CO_2_-induced anaesthesia, it causes paralysis or death in infected flies (Brun & Plus 1980). We genotyped all flies for the polymorphism in *ref*(*2*)*P* that is associated with resistance, identified females as *D. melanogaster* by polymerase chain reaction (PCR), and confirmed the infection status of flies with low transmission rates by reverse transcriptase-PCR (see the electronic supplementary material). Differences in transmission rate were analysed using generalized linear models with a quasi-binomial error distribution.

## 3. Results

We tested 900 wild-caught *D. melanogaster* for infection with the sigma virus. The prevalence was 10.3 per cent (95% binomial CI = 8.3–12.6%), and did not differ between females and males (*χ*^2^ = 0.33, *n*_female_ = 456, *n*_male_ = 444, d.f. = 1, *p* = 0.57). The mean female transmission rate was 83.3 per cent (95% CI = 71.2–93.1%), and mean male transmission was considerably lower at 46.9 per cent (95% CI = 35–59%), a difference that was highly significant (generalized linear model (GLM) with quasi-binomial error structure, *t*_79_ = 3.975, *n*_female_ = 39, *n*_male_ = 48, *p* < 0.001).

**Figure 1.**
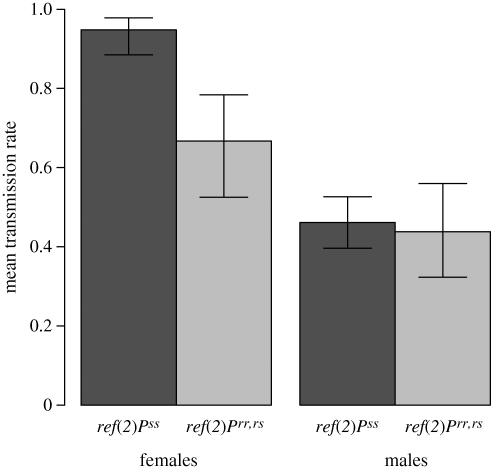
Effect of *ref*(*2*)*P* on the rate male and female flies transmit the sigma virus to their offspring. Flies with the resistant *ref*(*2*)*P* genotype (homozygous or heterozygous for the resistant allele) are shown in light grey and flies homozygous for the susceptible allele in dark grey. The means and standard errors were estimated using a GLM and back-transformed from the logit scale.

The resistant allele of *ref*(*2*)*P* was at a frequency of 24.0 per cent (*n* = 835), with 7.1 per cent of the population being homozygous for the resistant allele and 33.8 per cent heterozygous. As transmission rates are sex-specific, we analysed the sexes separately. We also pooled heterozygous and homozygous individuals carrying the resistant allele, as there were very few infected homozygous flies. The rate of female transmission was affected by the mother's genotype (GLM with quasi-binomial error structure, *t*_30_ = 2.096, 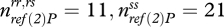, *p* = 0.04; figure 1), with transmission being on average 28.1 per cent lower in females that carried the resistant allele. Male transmission, on the other hand, was not affected by the father's genotype (GLM with quasi-binomial error structure, *t*_48_ = 0.156, 
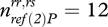
, 
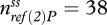
, *p* = 0.87, Δ_transmission_ = 2.3%; figure 1).

## 4. Discussion

Even robust results from association and linkage mapping studies are not always replicable in the field. Here, we have found that the results of laboratory-based linkage and association mapping for resistance to a virus are replicated in a wild population. This result is interesting as the association was replicated under uncontrolled field conditions and was robust to the genetic background of the resistant allele. Furthermore, the association was found in spite of a known genetic interaction between the parasite and the host genes. A genotype of the virus that overcomes *ref*(*2*)*P*-mediated resistance is common in European populations (Fleuriet & Sperlich 1992; Fleuriet & Periquet 1993).

The effect size of QTLs and polymorphisms identified by association mapping can vary and even differ in direction in males and females, a fact that has long been puzzling (Mackay *et al*. 2009). We found such a sex difference in this study, with a strong association in females but no effect in males. This difference can be explained by the underlying genetics. In the laboratory, the resistant allele of *ref*(*2*)*P* reduces both paternal and maternal transmission by similar amounts (Bangham *et al*. 2008*a*). But while transmission through females has a largely bimodal distribution, with *ref*(*2*)*P* explaining all of the genetic variation in this trait, male transmission is continuously distributed and affected by other genes (Bangham *et al*. 2008*a*,*b*). We found the same difference in the variation of male and female transmission in the field. In females the rates followed a bimodal distribution of mostly low (less than 7%) and high transmission rates (more than 77%), with only one individual with an intermediate value of 44 per cent infected offspring. Males on the other hand showed a nearly continuous distribution of transmission rates (data not shown; note that this pattern could be amplified by non-genetic factors under field conditions). These factors will reduce our power to detect associations in males.

The results from our study are encouraging for laboratory-based association and linkage mapping studies, showing that this approach can yield results that are directly replicable in the field. At the same time, they demonstrate that such replication in the field is feasible and that quantitative and evolutionary biologists should endeavour to validate results from laboratory experiments on genetic model systems in natural populations whenever possible. This is a crucial, but often overlooked, step in understanding the genetics of complex traits such as disease and parasite resistance.
